# Changes in Food Consumption Trends among American Adults since the COVID-19 Pandemic

**DOI:** 10.3390/nu15071769

**Published:** 2023-04-05

**Authors:** Lillie Monroe-Lord, Elgloria Harrison, Azam Ardakani, Xuejling Duan, Lily Spechler, Tia D. Jeffery, Phronie Jackson

**Affiliations:** 1 College of Agriculture, Urban Sustainability and Environmental Sciences, University of the District of Columbia, Washington, DC 20008, USA; 2 Lehman College, City University of New York, Bronx, NY 10468, USA; 3 Data Analytics, McDaniel College, 2 College Hill, Westminster, MD 21157, USA

**Keywords:** COVID-19, Americans adults, MyPlate, dietary habit, pandemic, food consumption, dietary screening tool

## Abstract

The quality and quantity of food consumption have changed due to the COVID-19 pandemic. In this study, we examined how the COVID-19 pandemic has changed the consumption of different food groups in order to close the research gap by providing current evidence that reflects a later stage of the pandemic compared to other circulating research conducted at earlier stages. Data collection for this cross-sectional study was performed via an online Qualtrics survey from 10,050 adults aged 40–100 years. Nutritional status was measured using the 24-item short-form Dietary Screening Tool (DST) twice: before and since the COVID-19 pandemic. The DST questions were categorized based on MyPlate items, along with fat, sugar, and sweet items, as well as nutritional supplement intake. In addition, the total DST score was calculated for each participant, which categorized them into one of three groups: “at risk”, “possible risk”, and “not at risk”. The results revealed that the consumption of grains, fruit, lean protein, and dairy decreased significantly, while the consumption of fat, sugar, and sweet items increased significantly due to COVID-19. The biggest decreases in consumption of food subcategories were related to whole grain bread and cereal, followed by fruit as a snack, in comparison with other types of grain and fruit. No changes in the consumption of vegetables, processed meat, or supplement intake were seen. The total DST score showed that, before and since COVID-19, the overall nutrition status of adult Americans has been at risk. In addition, of those participants who were not at risk before COVID-19, 28.5% were either at risk or at possible risk since COVID-19; moreover, of those participants who were at possible risk before COVID-19, 21% were at risk since COVID-19. As a good nutritional status can reduce the risk of severe illness or even mortality rate in times of crisis, the findings of this study can help policymakers and health educators to develop heath-protecting behavior sessions against future pandemics to manage crises.

## 1. Introduction

With so many unprecedented changes borne out of the COVID-19 pandemic, it is unsurprising that food consumption patterns have also changed. The pandemic has impacted the quality and quantity of people’s diets in many ways. Some studies have found that individuals had more time for cooking healthy meals and ended up reducing fast-food consumption upon staying at home [1,2,3], while the consumption of fruit and vegetables decreased overall [4]. Furthermore, others have recorded an increased intake of energy-dense snack foods due to psychological pressures during the pandemic [2,5,6,7,8]. However, the consumption of other food categories, including grains, protein, and dairy, has not been investigated since the COVID-19 pandemic.

It is well known that nutrition plays a role in maintaining a healthy immune system, with nutrient deficiencies being able to impair immunity [9]. It came as no surprise that nutrition status was a factor in illness severity during the COVID-19 pandemic. Due to the anti-inflammatory role of antioxidants in the diet, those who exhibited poor nutrient status (particularly in vitamin A, vitamin E, selenium, and vitamin C) were at higher risk of oxidative stress [10]. Vitamin D status is especially essential for protection against COVID-19 due to its role in modulating the inflammatory response and protecting individuals from acute respiratory distress and pneumonia [11,12]. Those who followed plant-based diets, consumed total vegetables, and plant proteins, as well as consuming less poultry, red, and processed meats, sugar-sweetened beverages, and alcohol, were found to have a 73% decreased risk of developing severe COVID-19, likely due to the protective role of plants and the inflammatory impact of many animal-based products [13]. Diets high in sugar were found to contribute to a proinflammatory state and worse outcomes of COVID-19 [14].

Among different populations, vulnerable individuals seem to have been impacted by the pandemic to a greater extent [15]. Older adults were more vulnerable compared to the younger population, with older age being linked to a higher risk of developing severe illness caused by COVID-19 because of physiological changes and increased comorbidities [16]. Older adults are also more vulnerable to social isolation, socioeconomic hardship, physical disability, reduced physical activity, and limited access to healthy food, which impacted their diet even before the pandemic [17].

Furthermore, the observed changes in dietary patterns appear to be dependent on the stage of the pandemic. At the beginning of the pandemic, the fear and uncertainty of the initial lockdowns led to an increase in “unusual purchasing” and “food hoarding,” with consumers being more prone to “stockpiling” behavior, resulting in empty shelves in grocery stores [18]. Most of the available literature up until this point has exposed dietary habits toward the beginning of the pandemic during the lockdown but there has not yet been much research about some of the longer-lasting implications of the COVID-19 pandemic.

The purpose of this study was to examine how the COVID-19 pandemic has changed the dietary habits of adults aged 40–100 years. The aim was to close the research gap by providing current evidence that reflects a later stage in the pandemic compared to other circulating research performed at the start of the lockdown. This study also considered all MyPlate food items to examine the changes in dietary habits among American adults, which was missing in the existing literature until now. The findings of this study can help policymakers to design and develop strategies to assist especially vulnerable populations to reduce the negative health impacts in cases of future crises such as the COVID-19 pandemic.

## 2. Method

### 2.1. Design, Participants, and Procedure

The design of this study was cross-sectional. The questionnaire was designed to determine the impact of the COVID-19 pandemic on nutritional status, physical activity, food security, and quality of life among vulnerable populations living in urban cities who were more likely to experience severe symptoms or die from COVID-19-related complications. The sample included 10,050 participants aged 40–100 years. Data were collected from 9 August to 15 September 2020 via an online Qualtrics survey. Qualtrics sought to collect a balanced sample in terms of demographic background, including sex (male and female), race (White, Asian, and Black), and ethnicity (Hispanic and non-Hispanic). The survey was completed under two conditions: “before” (a retrospective condition) and “since” the start of COVID-19 in the United States. More details of the survey were previously published [19].

### 2.2. Measure

Nutritional status was measured using the 25-item short-form Dietary Screening Tool (DST). This tool was developed and validated for use in middle-aged and older adult populations [20,21]. The questions differ in the number of response options. Each response option took score points resulting in a total score range of 0–100. Healthier options for each question received a higher score. Thus, higher scores indicate a healthier diet. Nutrition status was measured twice retrospectively: before and since the COVID-19 pandemic [19].

For assessing the dietary habits of the participants, the DST questions were categorized based on MyPlate items (i.e., fruit, vegetable, proteins—divided into lean protein and processed meat)—grain, and dairy), along with fat, sugar, and sweet (FSS) items and nutritional supplement intake [20,21,22]. In addition, the total DST score was calculated for each participant. According to the DST scores, our sample was categorized into three groups: “At risk” with a DST score less than 60, at “possible risk” with a DST score between 60 and 75, and “not at risk” with a DST score of more than 75 [20,21].

### 2.3. Software and Statistical Tests

The data were analyzed using R (version R 4.1.1). Descriptive statistics are provided as the mean and standard deviation (SD) for continuous variables, and as the frequency and percentage for categorical variables. Statistical significance was set at *p* < 0.05. Differences between nutritional items before and since COVID-19 were examined using a paired-samples *t*-test and the McNemar test. The McNemar test was used to assess the yes/no questions.

## 3. Results

The details of the sample’s characteristics are presented in Table 1. The participants were 42.6% male and 57.4% female. Approximately 60% of the participants were above 61 years of age with a mean age of 62.04 years. The majority of the participants (73.6%) were White, and more than half (51.7%) had a college degree or above.

The biggest difference in fruit item consumption was seen for fruit consumed as a snack (~6%), whereas the smallest difference was in juice consumption since the COVID-19 pandemic (*p* < 0.001). However, the consumption of different vegetables and their amounts has not changed since the COVID-19 pandemic. Consumption of chicken and turkey, non-fried fish, or seafood, which are considered lean proteins, significantly decreased (*p* < 0.001) and did not change (*p* = 0.093) due to COVID-19. Consumption of processed meat did not change during the pandemic. In addition, the consumption of milk as a sole source of dairy and the daily serving size of milk, cheese, or yogurt have decreased significantly since COVID-19 (*p* = 0.023 and *p* < 0.001, respectively). Grain consumption decreased for all items, with a greater change in whole grain bread consumption (11.5% decrease) since the COVID-19 pandemic. In terms of the consumption of sweets and snacks, candy and chocolate decreased by 4.8%; crackers, pretzels, chips, and popcorn significantly decreased by ~4.1%; cookies and ice cream decreased by 1.7% and 0.8%, respectively (*p* < 0.001 for all items). Consumption of fatty items, butter and margarine on bread, rolls, and biscuits, and gravy significantly decreased (*p* < 0.001). Consumption of fat on potatoes and other vegetables also decreased (*p* = 0.038). No change was seen in the consumption of sugar or honey to sweeten a participant’s coffee or tea. Consumption of alcoholic drinks has significantly decreased by 2.5% since COVID-19 (*p* < 0.001). However, nutritional supplement use did not significantly change among the participants, although consumption has increased since COVID-19 (Table 2).

The DST mean scores for fruit, grains, lean proteins, and dairy showed a significant decrease of 4.22%, 7.42%, 1.40%, and 1.40% since the COVID-19 pandemic, respectively (*p* < 0.001). However, the DST score for FSS indicated a significant increase of 3.64% since the COVID-19 pandemic (*p* < 0.001). The DST score for the consumption of vegetables and processed meats highlighted a non-meaningful change among participants since the COVID-19 pandemic (*p* = 0.383 and *p* = 0.276, respectively). The DST score of grain consumption showed the largest reduction by 7.42% in comparison to the score of other MyPlate food categories since the COVID-19 pandemic (*p* < 0.001).

The largest decrease in consumption since the COVID-19 pandemic was recorded for FSS items with approximately 39%, followed by grain and fruit consumption at 32% and 31%, respectively. In addition, the largest increase in consumption since COVID-19 was also recorded for FSS with approximately 23%, followed by vegetables and fruit with 20% and 18%, respectively (Table 3).

In the next part of the analysis, the total DST score was calculated for before and since the COVID-19 pandemic. The results revealed that the mean DST score before the pandemic was 56.04 (IQR: 48.00–65.00), with the mean score decreasing to 55.54 (IQR: 47.00–64.00).

We divided our sample population into three groups based on their DST scores: not at risk, at possible risk, and at risk. It is interesting that, of those participants who were not at risk before COVID-19, 28.5% have become either at risk or at possible risk since COVID-19. In addition, of those participants who were at possible risk before COVID-19, 21% are now at risk since COVID-19. Of those participants who were at risk before COVID-19, 90% have remained at risk since COVID-19 (Table 4).

## 4. Discussion

This study examined the dietary changes among adults since the COVID-19 pandemic in the United States. To date, there has been no study assessing the change in all MyPlate food items (i.e., fruit, vegetables, proteins, grains, and dairy), along with FSS items and nutritional supplement consumption, since the COVID-19 pandemic. This research helps in understanding how crisis situations and pandemics play a role in changing the dietary habits of the middle-aged and older adult populations. The findings of this study are vital for the development of strategies to improve and manage diet quality during future crises.

Fruit consumption has decreased since the COVID-19 pandemic. This finding is consistent with previous studies showing a reduction in fruit consumption [23,24]. A previous study reported that this decreasing trend is higher among food-insecure individuals than food-secure individuals [25]. This trend in fruit intake is problematic, especially during a pandemic, because fruit is positively associated with health outcomes due to the presence of antioxidants, phytochemicals, and fiber in fruit [26]. It is possible that fruit consumption decreased due to the heightened fear of germs since fruit is more commonly eaten raw, which may pose a higher risk of viral infections compared to cooked foods. It would be beneficial to pinpoint the reason(s) fruit consumption decreased so that future policies can support fruit intake during pandemics. In addition to the reduction in fruit consumption, juice consumption has significantly decreased since the pandemic. This finding is at odds with other studies in circulation, which showed that juice consumption, specifically orange juice consumption, significantly increased [27,28]. Heng et al. suggested that the motivation for this increase in orange juice consumption was due to a heightened fear of the virus, and the belief that orange juice can boost one’s immunity as it is a good source of vitamin C [27]. This inconsistency in findings about juice consumption might be due to the focus on the specific type of juice and the time of data collection. Previous studies focused on the early stages of the pandemic, while our research was conducted at a later stage of the pandemic when concerns about the virus may have dissipated.

Previous studies concerning vegetable consumption since the COVID-19 pandemic were inconsistent [4,29]. In our study, vegetable consumption did not change. One explanation may be related to the study sample, which was predominantly Caucasian. A previous study revealed that Caucasians in the United States tend to have the greatest access to fresh vegetables [30]. This comparatively stable pattern of vegetable intake may also be due to heightened concerns about immunity and wellness in the face of the virus. Due to financial uncertainties, with fewer available funds, it is also possible that individuals prioritized healthier choices such as vegetables. Furthermore, we postulate that there may have been less resistance to vegetable consumption in terms of viral transmission risk considering that cooking them is a common preparation method, especially among seniors who are more prone to dentition issues [31].

The largest reduction among MyPlate food items was in grain consumption since the COVID-19 pandemic; however, a study conducted in European countries revealed an increase in grain consumption [32]. The reduction in grain consumption in our study might be related to disruptions in the food supply chain, especially grain products, during the pandemic [33]. There were shortages of some food products such as wheat (e.g., flour) during the pandemic.

The change in protein consumption differed according to the type of protein. Lean protein consumption decreased, while processed meat consumption did not change following the COVID-19 pandemic. This variation, according to the type of protein, is consistent with a European study, which showed that seafood consumption as a source of protein was limited during the COVID-19 pandemic, whereas meat consumption did not change during this time [32]. According to the USDA, the price of meats, poultry, and cereal rose sharply by over 3% during this time [34]. A decreased consumption of meat, dairy, and grains might be reflective of reduced buying power. The meat industry also saw many supply chain disruptions. These supply chain disruptions were caused by the rapid spread of the virus among workers, decreases in the labor force, export issues, and changing legislation governing food exports [35]. These disruptions may also have played a role in the downward trend of meat consumption. However, our study found that the consumption of seafood has remained unchanged since the COVID-19 pandemic. One possible reason for this is that most Americans were not eating an adequate amount of seafood even prior to the COVID-19 pandemic, and they were more likely to get protein from meat than from seafood [36].

Total dairy consumption has decreased since the COVID-19 pandemic. This reduction can be attributed to the increasing price of dairy products due to the COVID-19 pandemic as well as supply chain disruptions [34,37]. However, some studies have also suggested an increase in dairy consumption, with this increased demand being a potential source of supply chain disruptions [32,38]. It is well documented that vitamin D deficiencies can contribute to severe cases of COVID-19, due to vitamin D’s role in modulating the immune system and increasing surfactant expression in the lungs [39,40,41,42,43]. Therefore, it is important to address ways to prevent decreases in dairy consumption during pandemics, since dairy is a significant source of dietary vitamin D. Research on post-lockdown dairy consumption is limited; therefore, future studies can further examine this gap to investigate the main reason for the reduction in dairy consumption.

FSS consumption, as an indicator of unhealthy snacking, was among the few food items that have increased significantly since the COVID-19 pandemic according to our findings. Most food items in this category constituted snacks eaten by participants. Previous studies stated no changes in unhealthy snack consumption, while others found a trend toward an increase in this food group [44]. For the groups that did increase their snacking habits, experts have suggested a link to increased psychological distress and uncertainty, a phenomenon known as “emotional eating” [45]. Increased snacking habits were also associated with individuals who already had a higher BMI [46,47].

We found that alcohol consumption has decreased by 2.6% since the COVID-19 pandemic. This finding is at odds with other studies, conducted during the early stages of lockdown, which showed a significant increase in alcohol consumption [44,48,49]. Increased alcohol consumption was associated with the heavy lockdown restrictions, while regions that had fewer restrictions did not experience the same increase [49]. Psychological distress and age were also contributing factors, with a greater increase in alcohol consumption associated with younger adults compared to more mature adults [50]. Our study may have differed from previous studies suggesting increased alcohol use during the pandemic because of our focus on the behaviors of older adults and the exclusion of young adults.

Interestingly, we found that the use of nutritional supplements has decreased since the COVID-19 pandemic. This finding contradicts previous studies that showed an increase in the intake of supplements [51]. According to a Market Researchers Report, there was a striking 44% increase in dietary supplement sales from 2019 to April 2020 [51]. Despite these trends in increased supplement use, the vast majority of supplements have no effect on disease outcomes, with the exception of vitamin D and zinc supplements [52]. This disparity in our findings might have been a result of the stage of the pandemic, our smaller population size, or our demographic of older adults on a fixed income compared to the larger database of supplement sales.

One advantage of our study is its large sample size of over 10,050 participants, with a focus on adults aged 40–100 years. Another advantage is that our sample demographic was similar to the greater population, except for the Hispanic population, which only represented 4% of our sample, compared to 18% of the greater U.S. population. Our study was conducted between August and September of 2020; this timeframe is unique in that it reflects the later stages of lockdown, while many other studies have focused on the immediate impact of the lockdowns. One limitation of this study is the possibility of recall bias associated with completing the survey for the questions regarding before the COVID-19 pandemic. Another disadvantage of this study is that it does not reflect post-lockdown America. Furthermore, the DST only considers specific vegetable items; thus, considering other vegetables with other instruments can help to precisely establish the estimated change in the consumption of this food item. Since our findings of decreased food consumption may reflect food insecurity following the pandemic, future studies can focus on continuing to track changes in dietary intake, even after the provision of vaccines enabled lockdowns to end.

Recent inflation and increased transportation costs could lead to further decreases in food consumption. One systematic review and meta-analysis found that increased food prices in any food group correlated with decreased consumption, especially in lower-income areas [53]. Moreover, considering subcategories of food in different demographic groups can also be examined in a future study, e.g., how the consumption of different sources of protein changed during the COVID-19 pandemic in different socioeconomic categories. Therefore, the findings of the study could precede the increased prevalence of diet-related chronic diseases without any substantial program/policy-related provisions that make healthy foods more accessible to vulnerable populations, which is even more vital during periods of global crises. Lastly, it is imperative for legislators to fully consider how food price inflation coupled with a reversion back to pre-COVID-19 SNAP benefits that were lower in monetary support than COVID-19-related SNAP benefits could be a deterrent to Health People 2030 goals.

## 5. Conclusions

This study focused on how the COVID-19 pandemic changed patterns in the consumption of different food items. The results revealed that the consumption of grains, fruit, lean proteins, and dairy decreased, while the consumption of unhealthy foods containing fat, sugar, and sweet items increased due to COVID-19. No changes in the consumption of vegetables, processed meat, or supplement intake were found in this study as a result of the COVID-19 pandemic. In general, the quality of nutrition has reduced since the pandemic among adult Americans, whereby almost one-third of those participants who were not at risk before COVID-19 have become either at risk or at possible risk since COVID-19.

## Figures and Tables

**Table 1:** Demographic characteristics of participants.

Characteristic	Frequency (n)	Percentage (%)
**Gender**		
Male	4274	42.6
Female	5761	57.4
**Age (years)**		
40–60	3864	38.4
61–80	5908	58.8
81–100	263	2.6
**Ethnicity/race**		
White	7381	73.6
African American	1393	13.9
Asian	698	7
Hispanic	431	4.3
Prefer not to answer	132	1.3
**Education**		
Less than and high school	1630	16.3
Some college	3207	32
College degree and more	5183	51.7
**Annual income (USD)**		
Less than 25,000	1547	15.4
25,000–49,999	2243	22.4
50,000–74,999	1893	18.9
75,000 or more	3944	39.3
Prefer not to answer	408	4.1

**Table 2:** Consumption of all items according to the DST before and since COVID-19.

	*n* (%)	Before Mean (SD) or *n* (%)	Since Mean (SD) or *n* (%)	MPC (%)	*p*-Value	*t*-Score	Effect Size (OR)
**Fruit items**							
How often do you usually eat fruit as a snack?		3.81 (1.49)	3.58 (1.56)	−6.264	<0.001	23.08	0.23
Less consumption since COVID-19	2193 (21.82)						
No change since COVID-19	7147 (71.05)						
More consumption since COVID-19	716 (7.12)						
How often do you eat fruit (not including juice)? Please include fresh, canned, or frozen fruit.		3.08 (1.71)	2.98(1.75)	−3.083	<0.001	7.76	0.077
Less consumption since COVID-19	1668 (16.60)						
No change since COVID-19	7061 (70.26)						
More consumption since COVID-19	1321 (13.14)						
How often do you drink some kind of juice at breakfast?		1.6 (1.92)	1.57 (1.57)	−1.5155	0.012	2.49	0.025
Less consumption since COVID-19	924 (9.19)						
No change since COVID-19	8308 (82.67)						
More consumption since COVID-19	818 (8.14)						
**Vegetable items**							
How often do you eat carrots, sweet potatoes, broccoli, or spinach?		5.48 (2.64)	5.48 (2.67)	0	1	0.00	0.00
Less consumption since COVID-19	1440 (14.33)						
No change since COVID-19	7120 (70.85)						
More consumption since COVID-19	1490 (14.83)						
How many different vegetable servings do you usually have with your main meal of the day?		2.81 (2.31)	2.83 (2.33)	0.7869	0.158	−1.41	−0.014
Less consumption since COVID-19	881 (8.77)						
No change since COVID-19	8221 (81.80)						
More consumption since COVID-19	948 (9.43)						
**Lean protein items**							
How often do you eat fish or seafood that is not fried?		1.94 (1.63)	1.93 (1.67)	−0.882	0.093	1.68	0.017
Less consumption since COVID-19	1167 (11.61)						
No change since COVID-19	7852 (78.13)						
More consumption since COVID-19	1031 (10.26)						
How often do you eat chicken or turkey?		3.27 (1.54)	3.21 (1.54)	1.715	<0.001	5.13	0.051
Less consumption since COVID-19	1042 (10.37)						
No change since COVID-19	8196 (81.55)						
More consumption since COVID-19	812 (8.08)						
**Processed meat items**							
How often do you eat cold cuts, hot dogs, lunchmeats, or deli meats?		3.35 (1.75)	3.33 (1.74)	−0.550	0.145	1.42	0.015
Less consumption since COVID-19	1043 (10.38)						
No change since COVID-19	7860 (78.21)						
More consumption since COVID-19	1147 (11.41)						
How often do you eat bacon or sausages?		3.85 (1.55)	3.85 (1.52)	−0.026	0.923	0.10	0.001
Less consumption since COVID-19	787 (7.83)						
No change since COVID-19	8425 (83.83)						
More consumption since COVID-19	838 (8.34)						
**Dairy items**							
How often do you drink a glass of milk?		1.29 (1.67)	1.27 (1.66)	1.463	0.023	2.28	0.023
Less consumption since COVID-19	946 (9.41)						
No change since COVID-19	8209 (81.68)						
More consumption since COVID-19	895 (8.91)						
How many servings of milk, cheese, or yogurt do you usually have each day?		3.21 (1.49)	3.17 (1.52)	1.360	<0.001	3.87	0.039
Less consumption since COVID-19	1061 (10.56)						
No change since COVID-19	8075 (80.35)						
More consumption since COVID-19	914 (9.09)						
**Grain items**							
How often do you usually eat whole-grain bread?		2.82 (2.00)	2.49 (1.95)	−11.552	<0.001	24.52	0.245
Less consumption since COVID-19	1895 (18.86)						
No change since COVID-19	7552 (75.14)						
More consumption since COVID-19	603 (6.00)						
How often do you usually eat whole grain cereal?		2.26 (2.04)	2.12 (1.99)	−6.289	<0.001	10.8	0.108
Less consumption since COVID-19	1332 (13.25)						
No change since COVID-19	7905 (78.66)						
More consumption since COVID-19	813 (8.09)						
How often do you eat hot or cold breakfast cereal?		2.42 (1.79)	2.33 (1.75)	−3.366	<0.001	8.72	0.087
Less consumption since COVID-19	1716 (17.07)						
No change since COVID-19	7195 (71.59)						
More consumption since COVID-19	1139 (11.33)						
**Fat, sugar, and sweet items**							
How often do you usually eat candy or chocolate?		2.04 (0.94)	2.21 (0.97)	8.167	<0.001	−19.07	−0.19
Less consumption since COVID-19	2028 (20.18)						
No change since COVID-19	7169 (71.33)						
More consumption since COVID-19	853 (8.49)						
How often do you eat crackers, pretzels, chips, or popcorn?		2.93 (0.87)	2.81 (0.9)	6.922	<0.001	−13.09	0.14
Less consumption since COVID-19	1867 (18.58)						
No change since COVID-19	7080 (70.45)						
More consumption since COVID-19	1103 (10.98)						
How often do you eat cakes or pies?		2.17 (0.77)	2.08 (0.81)	3.684	<0.001	−14.03	0.158
Less consumption since COVID-19	1728 (17.19)						
No change since COVID-19	7471 (74.34)						
More consumption since COVID-19	851 (8.47)						
How often do you eat cookies?		2.4 (0.86)	2.36 (0.9)	1.789	<0.001	−5.83	0.072
Less consumption since COVID-19	1484 (14.80)						
No change since COVID-19	7456 (74.19)						
More consumption since COVID-19	1107 (11.01)						
How often do you eat ice cream?		2.4 (0.85)	2.38 (0.9)	0.720	<0.001	−2.36	0.044
Less consumption since COVID-19	1289 (12.83)						
No change since COVID-19	7694 (76.56)						
More consumption since COVID-19	1067 (10.62)						
Do you usually add butter or margarine to foods like bread, rolls, or biscuits? Yes		7567 (75.4)	7400 (73.7)	−2.21	<0.001		0.605
Less consumption since COVID-19	426 (4.24)						
No change since COVID-19	9366 (93.19)						
More consumption since COVID-19	256 (2.57)						
Do you usually add fat (butter, margarine, or oil) to potatoes and other vegetables? Yes		6990 (69.7)	6932 (69.1)	−0.83	0.038		0.857
Less consumption since COVID-19	407 (4.05)						
No change since COVID-19	9296 (92.50)						
More consumption since COVID-19	347 (3.45)						
Do you use gravy (when available) at meals? Yes		5080 (50.6)	4977 (49.6)	−2.03	0.001		0.708
Less consumption since COVID-19	355 (3.53)						
No change since COVID-19	9444 (93.97)						
More consumption since COVID-19	251 (2.50)						
Do you usually add sugar or honey to sweeten your coffee or tea? Yes		4116 (41.0)	4116 (41.0)	0	1		1
Less consumption since COVID-19	254 (2.53)						
No change since COVID-19	9543 (94.96)						
More consumption since COVID-19	253 (2.52)						
Do you usually drink wine, beer, or other alcoholic beverages? Yes		4833 (48.2)	4709 (46.9)	−2.75	<0.001		0.648
Less consumption since COVID-19	353 (3.51)						
No change since COVID-19	9468 (94.21)						
More consumption since COVID-19	229 (2.28)						
**Supplement intake**							
Which of the following best describes your nutritional supplement use?		6885 (68.6)	6901 (68.8)	−1.21	0.427		1.09
Less consumption since COVID-19	170 (1.69)						
No change since COVID-19	9223 (91.77)						
More consumption since COVID-19	187 (1.86)						
Change in the type of supplement	470 (4.68)						

**Table 3:** Change in DST score and number (percentage) of each MyPlate food category due to the COVID-19 pandemic.

	*n* (%)	Before Mean (SD)	Since Mean (SD)	MPC ^1^ (%)	*p*-Value	*t*-Score	Effect Size
**Fruit**							
		8.49 (3.7)	8.13 (3.84)	−4.218	<0.001	17.542	0.175
Less consumption since COVID-19	3144 (31.28)						
No change since COVID-19	5091 (50.66)						
More consumption since COVID-19	1815 (18.06)						
**Grains**							
		7.5 (4.52)	6.95 (4.58)	−7.418	<0.001	22.734	0.227
Less consumption since COVID-19	3231 (32.15)						
No change since COVID-19	5250 (52.24)						
More consumption since COVID-19	1569 (15.61)						
**Vegetables**							
		8.29 (4.12)	8.32 (4.22)	0.267	0.383	−0.873	−0.009
Less consumption since COVID-19	1803 (17.94)						
No change since COVID-19	6271 (62.40)						
More consumption since COVID-19	1976 (19.66)						
**Lean proteins**							
		5.21 (2.54)	5.14 (2.52)	−1.405	<0.001	4.680	0.047
Less consumption since COVID-19	1775 (17.66)						
No change since COVID-19	6769 (67.35)						
More consumption since COVID-19	1506 (14.99)						
**Dairy**							
		4.5 (2.58)	4.44 (2.6)	−1.39	<0.001	4.323	0.011
Less consumption since COVID-19	1646 (16.38)						
No change since COVID-19	6880 (68.46)						
More consumption since COVID-19	1524 (15.16)						
**Fats, sugars, and sweets**							
		13.67 (4.46)	14.17 (4.67)	3.644	<0.001	4.323	0.043
Less consumption since COVID-19	3968 (39.48)						
No change since COVID-19	3748 (37.29)						
More consumption since COVID-19	2334 (23.22)						
**Processed meats**							
		7.2	7.18	−0.27	0.276	1.089	0.011
Less consumption since COVID-19	1470 (14.63)						
No change since COVID-19	6972 (69.37)						
More consumption since COVID-19	1608 (16.00)						

^1^ Mean percentage change (%).

**Table 4:** Nutritional risk based on DST score categories due to the COVID-19 pandemic.

Nutritional Risk	Since COVID-19			
	Not at Risk	Possible Risk	At Risk	<0.0001
Before COVID-19				
Not at risk	492 (71.51)	180 (26.16)	16 (2.33)	
At possible risk	182 (4.94)	2740 (74.44)	759 (20.62)	
At risk	8 (0.14)	562 (9.89)	5111 (89.97)	

## Data Availability

Data used during the current study are available from the corresponding author.
